# Power and sample size of two-stage extreme phenotype sequencing design for next generation sequencing studies

**DOI:** 10.1186/1471-2105-14-S17-A16

**Published:** 2013-10-22

**Authors:** Guolian Kang

**Affiliations:** 1 Department of Biostatistics, St. Jude Children’s Research Hospital, Memphis, TN 38105, USA

## Background

Next-generation sequencing technology is changing the genomic research due to its huge sequencing capacity which is used to identify rare susceptibility variants that affect complex diseases. Because of its high cost, a two-stage extreme phenotype sequencing (TS-EPS) design is an alternative approach [1] in which the genetic variants are discovered by whole-genome or whole-exome sequencing individuals with extreme phenotypes in stage I and the association variants are detected by sequencing a large number of individuals on the discovered variants in stage II. TS-EPS can efficiently discover more than half of the causal variants using about 0.2% of all individuals [2] and can therefore have higher power than random sampling given the sample size and effect sizes of the causal variants [2,3]. Using simulated data for unrelated individuals, we further explore the efficiency of TS-ESP in term of different sample sizes and varying effect sizes.

## Results

**Figure 1:**
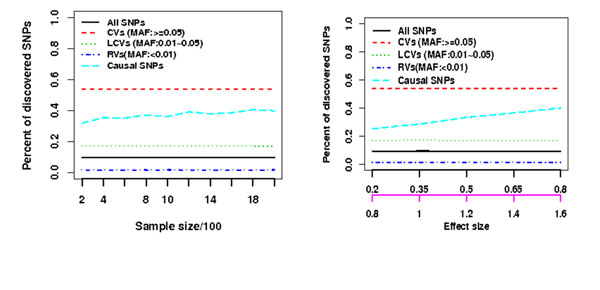
Stage I SNP discovery results for independent SNPs based on 4 individuals with EP.

**Figure 2:**
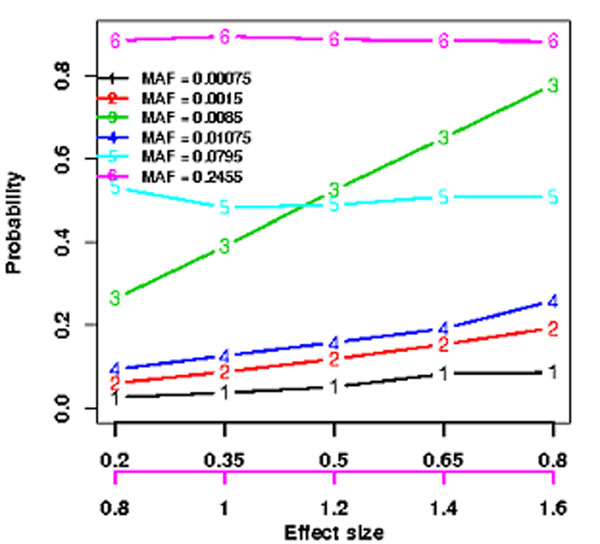
Enrichment of causal SNPs in 4 individuals with EP.

**Figure 3:**
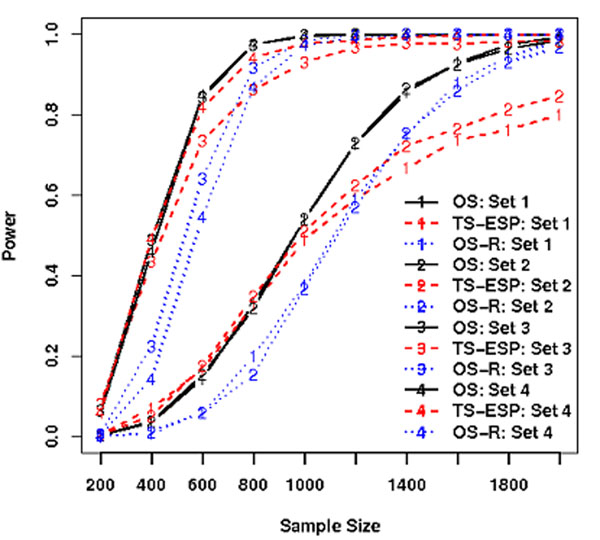
Power of TS-EPS for independent SNPs based on 4 individuals at a nominal level of 5×10^-5^. OS: one-stage design; TS-ESP: two-stage extreme phenotype sequencing design; OS-R: one-stage design exclude 4 individuals.

When four individuals with the first four most extreme trait values are sequenced in stage I, we found that 1) TS-EPS can discover the constant numbers of CVs, LCVs, and RVs regardless of the sample size and effect size, however the increasing numbers of causal variants with increasing sample size and effect size (Figure 1); 2) the probability of discovering a causal CV is constant regardless of its effect sizes but the quantity depends on its minor allele frequency; however, the probability of discovering RVs is a complex function of their effect sizes and minor allele frequencies given disease model and sample size (Figure 2). 3) Therefore, using an optimal unified association test for gene-based association analyses, the power of TS-ESP is comparable to one-stage (OS) design in which all individuals are sequenced for association testing if the rare causal variants have large effect sizes (Figure 3).
